# Transcriptome Analysis Reveals New Insights into the Modulation of Endometrial Stromal Cell Receptive Phenotype by Embryo-Derived Signals Interleukin-1 and Human Chorionic Gonadotropin: Possible Involvement in Early Embryo Implantation

**DOI:** 10.1371/journal.pone.0064829

**Published:** 2013-05-22

**Authors:** Amélie Bourdiec, Ezequiel Calvo, C. V. Rao, Ali Akoum

**Affiliations:** 1 Endocrinologie de la reproduction, Département d′obstétrique, gynécologie et reproduction, Centre de recherche du CHU de Québec, Hôpital Saint-François d′Assise, Québec, Québec, Canada; 2 Département de médecine moléculaire, Centre de recherche du CHU de Québec, CHUL, Faculté de médecine, Université Laval, Québec, Québec, Canada; 3 Department of Cellular Biology and Pharmacology, Herbert Wertheim College of Medicine, Florida International University, Miami, Florida, United States of America; Université de Technologie de Compiègne, France

## Abstract

The presence of the conceptus in uterine cavity necessitates an elaborate network of interactions between the implanting embryo and a receptive endometrial tissue. We believe that embryo-derived signals play an important role in the remodeling and the extension of endometrial receptivity period. Our previous studies provided original evidence that human Chorionic Gonadotropin (hCG) modulates and potentiates endometrial epithelial as well as stromal cell responsiveness to interleukin 1 (IL1), one of the earliest embryonic signals, which may represent a novel pathway by which the embryo favors its own implantation and growth within the maternal endometrial host. The present study was designed to gain a broader understanding of hCG impact on the modulation of endometrial cell receptivity, and in particular, cell responsiveness to IL1 and the acquisition of growth-promoting phenotype capable of receiving, sustaining, and promoting early and crucial steps of embryonic development. Our results showed significant changes in the expression of genes involved in cell proliferation, immune modulation, tissue remodeling, apoptotic and angiogenic processes. This points to a relevant impact of these embryonic signals on the receptivity of the maternal endometrium, its adaptation to the implanting embryo and the creation of an environment that is favorable for the implantation and the growth of this latter within a new and likely hostile host tissue. Interestingly our data further identified a complex interaction between IL1 and hCG, which, despite a synergistic action on several significant endometrial target genes, may encompass a tight control of endogenous IL1 and extends to other IL1 family members.

## Introduction

Embryonic implantation and establishment of successful pregnancy require a dynamic process of interactions between the embryo and a receptive maternal endometrium. This embryo/maternal cross-talk involves an elaborate and coordinated network of communication via timely released embryonic and maternal-derived signals and well-targeted actions. Optimal receptivity of the human endometrium to the implantation of a competent blastocyst occurs during a limited period of time within the menstrual cycle called “implantation window”, which is generally believed to span d6–10 following luteinizing hormone (LH) peak in the normal menstrual cycle [1], [2]. Numerous studies showed major and specific changes arising within this specific time interval, which encompass adhesion, invasion, survival, growth, differentiation and immune-modulating factors that shape up endometrial receptivity. The dynamics of this transition from a non-receptive to a receptive endometrium are poorly understood, but the correct spatio-temporal synthesis and balance of various factors is thought to play an important role in human uterine preparation for implantation [3], [4], [5].

Indeed, under the influence of a developing embryo, endocrine factors, particularly ovarian hormones, play a critical role in the regulation of the molecular changes that occur. Embryonic human chorionic gonadotropin (hCG) maintains for instance the production of progesterone by the corpus luteum, which is critical to sustain early pregnancy. However, direct interactions at the fetal-maternal interface and appropriate coordination between embryonic and maternal signals at the implantation site are essential for providing the synergistic environment needed for the establishment of pregnancy [6], [7].

hCG is a major embryonic signal playing a key role in the initiation and maintenance of pregnancy [8]. It is transcribed as early as the 2-cell embryo stage [9] and is produced abundantly by the trophectodermal cells of the pre-implantation blastocyst [10]. Following implantation, hCG is produced by syncytiotrophoblast of the developing conceptus [11]. Recent evidence suggests that hCG is also produced in glandular and luminal epithelium of human endometrium, primarily during the secretory phase [12], [13]. hCG production by embryonic cells may directly regulate the expression of endometrial factors and extend the period during which the endometrium is receptive [2], [14].

hCG acts on the intrauterine environment via the luteinizing hormone (LH)/hCG receptor (hLHCGR), which was detected in various cell types including human uterus and decidua, placenta and fetal membranes [15], [16]. Synthesized early by the trophoblast, hCG may therefore have a wide spectrum of cell targets and biological actions that influence endometrial receptivity and embryo implantation. It promotes human endometrial stromal cell (ESC) decidualization [17] via functional differentiation resulting in an up-regulation of cyclooxygenase 2 (COX2) gene expression and increased production of prostaglandin (PG)E_2_ [18], possesses both direct and indirect angiogenic properties [19], induces tissue specific human uterine natural killer (uNK) cell proliferation [20] and regulates embryonic autocrine and maternal paracrine factors involved in embryo attachment, endometrial remodeling, antioxidant defense and immune mechanisms around the implanting blastocyst [2], [21], [22].

Several studies provide strong evidence that interleukin (IL1)B may play a pivotal role at the embryo-maternal interface and represents one of the earliest signals [23], [24], [25], [26], [27]. IL1 is synthesized by the human embryo during its initial stages, and the concentration of this cytokine has been positively correlated with successful implantation after *in vitro* fertilization and transfer to the uterine cavity [28], [29]. A key regulator of the inflammatory response, IL1 is currently recognized as a multifunctional cytokine with a wide spectrum of effects on numerous cell types (eg nervous system cells, immune cells, connective tissue cells, endometrial cells, hepatocyte, fibroblast and endothelial cells) [30], [31]. IL1 acts on human endometrial cells to induce the secretion of leukemia inhibitory factor (LIF) and PGE_2_ [32], [33], which play an important role in the implantation process [34], up-regulates the expression of integrin β3, a marker of uterine receptivity, in human endometrial epithelial cells [35] and stimulates the migration of human first-trimester villous cytotrophoblast cells via endometrium-derived factors [36]. Purified human cytotrophoblasts in culture release IL1B in the manner that parallels their invasive potential [37]. IL1B stimulates the release of human placental metalloproteinase (MMP)9 [37], proMMP3 expression in baboon stromal cells [38] and hCG by first trimester human trophoblastic cells [39].

The IL1 system is composed of two receptors (IL1R1 and IL1R2), one accessory protein (IL1 RAP) also called IL1R3, one receptor antagonist (IL1RN) and two agonists (IL1A and IL1B), which both trigger cell activation via the functional signaling IL1R1 [40]. IL1R2 rather acts as a negative regulator of IL1 action. Either the membrane-bound (mb) or the soluble (s) form of IL1R2, which is released by proteolysis from the cell surface, acts by capturing IL1, thereby inhibiting IL1-mediated cell activation [41].

Our previous studies pointed to new mechanisms by which the embryo may fine-tune the receptivity of the maternal endometrium, and revealed the ability of hCG to interact with different human endometrial cell types and modulate cell receptivity to IL1. Actually, hCG appeared to down-regulate the expression of the inhibitory IL1R2 in endometrial epithelial cells without affecting that of the activating IL1R1 [42]. Comparable effects were observed in ESCs during the implantation window with, interestingly, a concomitant up-regulation of IL1R1, a down-regulation of IL1RN, an increased angiogenic activity and a higher secretion of monocyte chemotactic protein1 (MCP1) [23]. First identified as a specific factor for macrophage recruitment and activation, MCP1 was later found to be endowed with various immune modulating, proangiogenic and growth-promoting properties [43], [44]. The aim of the present work was to gain a broader understanding of the global impact of hCG on the modulation of human endometrial cell responsiveness to IL1 and the acquisition of growth-promoting phenotype capable of sustaining active embryonic implantation and growth. Using micro-array analysis of hCG, IL1 and hCG/IL1-treated ESCs from the implantation window, our data identified several significantly regulated genes targeted by hCG/IL1 synergy and implicated in angiogenesis, proliferation, tissue remodeling, cell signaling and immune modulation, which is relevant to early embryo implantation process, and a wide spectrum of targets encompassing IL1 family members.

## Materials and Methods

### Subjects and tissue handling

Endometrial tissue specimens were obtained during the implantation window (days 19 to 24) from normal fertile women with a regular menstrual cycle, who were undergoing laparoscopy for tubal ligation and had not received hormonal or anti-inflammatory therapy for at least 3 months prior to surgery (mean age ± SD, 35.6±4.9 yr.; *n* = 7). Menstrual cycle day was determined according to the histological criteria of Noyes *et al* [45]. A written informed consent was obtained from participants under a study protocol approved by the Ethics Committee on Human Research of Laval University, Quebec, Canada. Collection of endometrial tissue biopsies was performed using a Pipelle (Unimar Inc., Prodimed, Neuilly-En-Thelle, France). Tissue samples were kept at 4°C in sterile Hank's balanced salt solution (HBSS) containing 100 U/mL penicillin, 100 µg/mL streptomycin and 0.25 µg/mL amphotericin B (Invitrogen Life Technologies, Burlington, ON, Canada) and immediately transported to the laboratory.

### Cell culture and treatment

ESCs were isolated and characterized according to our previously described procedure [46]. Concisely, tissue was minced into small pieces, dissociated with collagenase before ESCs were separated by differential sedimentation and adhesion. The purity of primary ESC cultures was tested morphologically by light microscopy and immunocytochemically on parallel cultures, as previously described. Cultures were free of CD45^−^positive leukocytes and contamination by factor VIII-positive endothelial cells was generally less than 1%. ESCs were cultured at 37°C in DMEM∶F12 (1∶1) supplemented with 10% fetal bovine serum (FBS), insulin, transferrin, and a mix of antibiotics–antimycotics. Preconfluent cells were washed with HBSS, incubated overnight with charcoal-treated FBS-supplemented medium, washed with phenol red-free DMEM∶F12 (1∶1) and cultured with phenol red- and FBS-free medium containing hCG (100 ng/mL, recombinant protein expressed in a mouse cell line, 10,000 IU/mg; Sigma-Aldrich Co., St. Louis, MO) for 24 h. Cells were then incubated with a fresh phenol red- and FBS-free medium containing IL1B (0.1 ng/mL, R&D Systems, Minneapolis, MN) for additional 24 h. hCG and IL1B concentrations were determined based on our previous studies with human ESCs where different doses were used (Bourdiec A et al, Biol Reprod, 2012). hCG and IL1B concentrations are within the range of the molecules' physiological concentrations [28], [47].

### RNA preparation and micro-array analysis

Total RNA of ESC cultures issued from 3 different women was extracted with Trizol according to the manufacturer's directions (Invitrogen). Then they were washed using the micro RNeasy Kit (Quiagen). Total RNA quantity was measured with Nanodrop ND-1000 spectrophotometer (Nanodrop Technologies Inc., DE,USA) and RNA integrity was assessed by capillary electrophoresis using the Bioanalyzer 2100 (Agilent Technologies, Mississauga, Ontario, Canada). DNA micro-array analyses were carried out with Affymetrix Human Gene 1.0 ST at the Gene Expression Platform of the Research Centre of Laval University Hospital Centre, Quebec, Canada. The array interrogates 28,869 well-annotated genes with 764,885 distinct probes. The design of the Human Gene 1.0 ST Array was based on the March 2006 human genome sequence assembly (UCSC Hg18, NCBI build 36) with comprehensive coverage of RefSeq, Ensembl and putative complete CDS GenBank transcripts. Chips were processed according to the Affymetrix standard protocol. Briefly, total RNA (150 ng per sample) was labeled using the Ambion WT Expression kit and Affymetrix GeneChip® WT Terminal Labeling kit, and hybridized to the arrays as described by the manufacturer (Affymetrix, Santa Clara, CA). The cDNA hybridization cocktail was incubated overnight at 45°C while rotating in a hybridization oven. After 17±1 h of hybridization, the cocktail was removed and the arrays were washed and stained in an Affymetrix GeneChip fluidics station 450, according to Affymetrix protocol (http://media.affymetrix.com/support/downloads/manuals/wt_sensetarget_label_manual.pdf). The arrays were scanned using the Affymetrix GCS 3000 7G and the Affymetrix Expression Console Software (Affymetrix, Santa Clara, CA), to produce the intensity files.

Data analysis, background subtraction, and intensity normalization were performed using Robust Multiarray Analysis [48]. Differentially expressed genes and false discovery rate were estimated from t test (P<0.05) and corrected using Bayes approach [49]. Data analysis, hierarchical clustering, and ontology was performed using the OneChanelGUI to extend affylmGUI graphical interface capabilities [50] and Partek Genomics Suite, version 6.5 (Partek Inc., St. Louis, MO) with analysis of variance analysis.

### Quantitative real time PCR

RNA was extracted using the standard Trizol® protocol (Invitrogen) as previously reported _ENREF_36[51]. Quantitative real time (qRT)-PCR was performed on the same 3 cultures used for microarray analysis and 4 more different cultures. An ABI 7000 Thermal Cycler (Applied Biosystems, Foster City, CA) was used. Each standard PCR reaction contained 2 µL reverse transcriptase (RT) product, 0.5 µL of primer (final concentration, 0.1 mM), 12.5 µL of SYBR Green PCR Master Mix (Invitrogen) consisting of *Taq* DNA polymerase reaction buffer, *Taq* DNA polymerase, SYBR green I, deoxynucleotide triphosphate mix and MgCl_2_. The reaction melting temperature (Tm) and the list of primers are reported in the Table 1. Primers were designed with Primer Premier 5 software to cross intron-exon boundaries. All samples were tested in duplicate and for each reaction negative controls without RNA and without reverse transcriptase were included.

**Table 1:** List of PCR primers.

Gene	Primer	Tm°C
CCL2	F-CTCTGCCGCCCTTCTGT	60
	R-CTTCTTTGGGACACTTGCTG	
CCL5	F-CTCGCTGTCATCCTCA	56
	R-CACTTGCCACTGGTGTA	
CCL7	F-GCCTCTGCAGCACTTCTGTG	60
	R-CACTTCTGTGTGGGGTCAGC	
CCL8	F-CTTCAAGACCAAACGG	52
	R-GAATCCCTGACCCAT	
GAPDH	F- CAGGGCTGCTTTTAACTCTGG	60
	R-TGGGTGGAATCATATTGGAACA	
IL18R1	F-CTGGAGGAGCTGTTGT	60
	R-GATTAGTCTTCGGCTTT	
IL1A	F-AAGACAGTTCCTCCAT	52
	R-TTGCTACTACCACCAT	
IL1B	F-ACAGTGGCAATGAGGATG	58
	R-TGTAGTGGTGGTCGGAGA	
IL1RL1	F-CTGAGGACGCAGGTGA	54
	R-CTCCGATTACTGGAAACA	
IL18	F-GCCAGCCTAGAGGTATG	60
	R-GTTATCAGGAGGATTCATTT	
IL33	F-CAGGTGACGGTGTTG	56
	R-TGTAGGACTCAGGGTTA	
IL6	F-GGAGACTTGCCTGGTGAA	60
	R-GCATTTGTGGTTGGGTCA	
KRT19	F-CGACAATGCCCGTCTG	58
	R-GCCTGTTCCGTCTCAAA	
MMP10	F-CAAGAGGCATCCATAC	54
	R-AACCTTAGGCTCAACT	
MMP9	F-TTGACAGCGACAAGAAGTGG	54
	R-CCCTCAGTGAAGCGGTACAT	
PTGS2	F-TCCCTTGGGTGTCAAAGGTAA	60
	R-AAAACTGATGCGTGAAGTGCTG	
TIMP3	F-CTCCGACATCGTGATC	54
	R-TCCTTTACCAGCTTCTT	
VCAM1	F-TGAAGGATGCGGGAGT	58
	R-GCAGGTATTATTAAGGAGG	
VEGFC	F-GCCAGCAACACTACCA	58
	R-TTGAGTCATCTCCAGCAT	

### Enzyme-Linked Immunosorbent Assay (ELISA)

CCL2 and CCL5 concentrations in the culture medium were measured using previously reported sandwich ELISAs [52], [53]. VEGFC, TIMP3, MMP9 and prolactin were measured using DuoSet kit (DuoSet, R&D Systems, Minneapolis, MN), according to the manufacturer's instructions.

### Statistical analysis

qRT-PCR and ELISA data followed a parametric distribution and were expressed as means ± SEM. Statistical analyses were performed with GraphPad Software Prism 4.0 (GraphPad Software, Inc., San Diego, CA, USA). The significance of statistical differences was determined using one way analysis of variance (ANOVA) followed by the Bonferroni test *post hoc*, for multiple comparisons, and the Student's t-test for the comparison of two groups.

## Results

### Gene expression profile for all experiments

**Figure 1:**
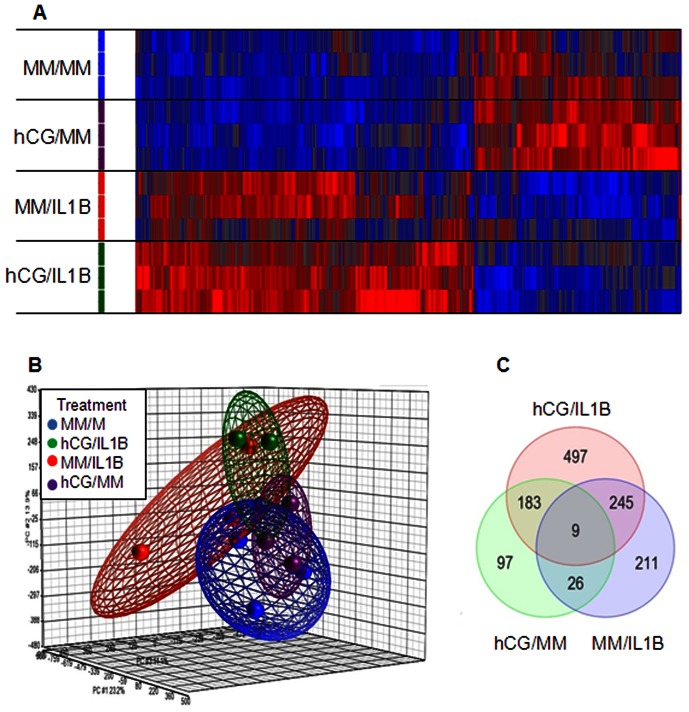
Analysis of genes significantly modulated by each treatment. A) Headmap of probe sets corresponding to genes significantly modulated (P<0.05) in each group. Increased signal intensities are displayed in red, whereas lower signal intensities are shown in blue. Cluster distances were evaluated by Spearman correlation on average linkage (Partek Genomics Suite). B) PCA scatter plot of all samples was generated to assess the variability of micro-array data. Each sphere represents a whole chip data. As shown in the legend, samples are colored by treatment and grouped by an ellipsoid that considers two standard deviations from the center of each group. C) Venn diagram of the respective gene lists showing the overlap of action between hCG/MM, MM/IL1B and hCG/IL1B. Data were obtained with ESC cultures issued from 3 different subjects.

Total RNA was isolated from primary human ESCs treated with hCG (100 ng/mL) for 24 h before being stimulated or not with IL1B (0.1 ng/mL) for additional 24 h. RNA from all treated groups was compared by micro-array with the corresponding non-treated control using Affymetrix GeneChip Human Genome. Using Significance Analysis of Micro-arrays (SAM), all genes significantly regulated between treated versus the non-treated group were selected [fold change (FC) 1.5 and a false discovery rate (FDR) <5%]. The unsupervised hierarchical clustering analysis of the array data showed specific molecular signatures of the global gene expression for each group and a noticeable discrimination between IL1B-treated and IL1B-untreated cells with and without hCG pre-treatment (Figure 1A). Three dimensional Principal Component Analysis (PCA) further showed different patterns of gene expression and a clear segregation between the four groups included in this study [MM/MM (control minimal medium), hCG/MM, MM/IL1B and hCG/IL1B treatments). Also, samples from the same group were very tightly clustered together, which corroborates the robustness of the Affymetrix micro-arrays (Figure 1B).

### Gene expression profile in ESCs is under embryonic stimuli

The different gene lists identified using SAM analysis (FC 1.5 and an FDR <5%) of treatment versus control groups were then intersected to determine their overlap. The results showed that 9 significantly regulated genes were common to all treatments, and 97, 211 and 497 genes were independently regulated by hCG, IL1B and hCG/IL1, respectively (Figure 1C), thereby suggesting highly specific expression profiles.

### Genes ontology (GO) of biological processes

**Figure 2:**
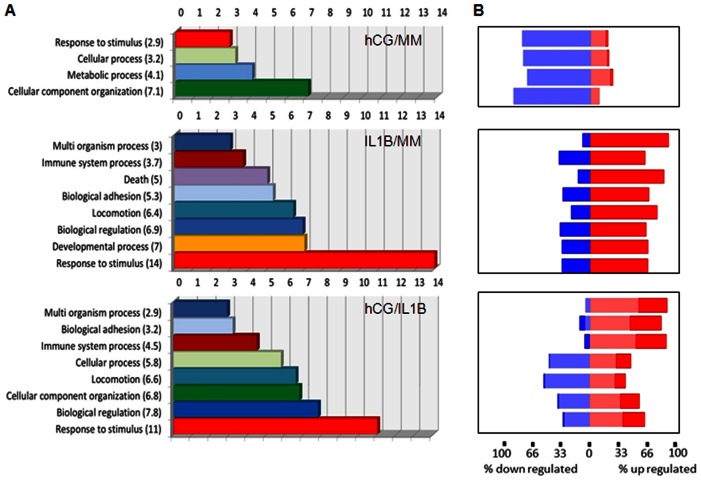
Enrichment score of biological processes. A) GO analysis was used to identify the main biological processes targeted by gene lists and significantly modulated by each treatment. Each functional group was assigned with a GO enrichment score that was calculated using a chi2 test. B) A forest plot using the same gene list was also generated to show the percentage of differentially expressed genes that were up-regulated (red) or down-regulated (blue) for each biological process. Light color: gene populations with a fold change ranging from 1.5 to 2. Dark color: gene populations with a fold change above 2. Data were obtained with ESC cultures issued from 3 different subjects.

Gene ontology (GO) annotations were then used to explore the specific functional properties of the molecular signatures. A functional enrichment analysis was performed using Partek software (Figure 2). Only significant biological functions were reported. The molecular signature of hCG/MM-treated group was enriched in genes associated with the regulation of cell response to stimulus, cellular and metabolic processes and cellular component organization. Analysis of the IL1B-treated group identified several enriched GO categories that were linked to multi-organism process, immune system process, death, biological adhesion, locomotion, biological regulation, developmental process and response to stimulus, with a marked increase in the latter process. In hCG/IL1 treated group, most enriched GO categories were similar to those found in the IL1B-treated group, but the enrichment scores appeared to be different. The most perceptible increase over hCG and IL1B/hCG was related to cellular, biological regulation and immune system processes, whereas other processes such as biological adhesion and response to stimulus seemed to be lessened to some extent. As observed in the forest plot shown in Figure 2B, IL1B increased the percentage of differentially up- and down- regulated gene populations with a fold change above 2 in each of these biological processes, while co-exposure to hCG led globally to a more moderated regulation. These observations are noteworthy considering the possible involvement of these critical biological processes in the embryo-maternal crosstalk and the establishment of pregnancy.

### Identification of differentially expressed genes implicated in early embryo implantation

Tables 2–5 summarize significantly up- and down-regulated genes in ESCs in response to hCG, IL1 and hCG/IL1 compared to untreated control cells. Genes were ranked according to the average of the fold change. To validate changes in the level of RNA transcripts, we have selected some genes found to be significantly regulated upon hCG, IL1B or hCG/IL1B treatment by micro-array and known for being involved in proliferation, immune modulation, tissue remodeling, cell signaling, apoptosis and angiogenesis, which are crucial for early embryo implantation. qRT-PCR was performed for the MCPs [chemokine C-C motif ligand (CCL)2 or MCP1, CCL8 or MCP2 and CCL7 or MCP3], vascular cell adhesion molecule 1(VCAM1), IL6, CCL5 or regulated and normal T cell expressed and secreted (RANTES), PG synthase (PTGS)2 or COX2, vascular endothelial growth factor C (VEGFC), MMP9, tissue inhibitor of metalloproteinase 3 (TIMP3) and keratin 19 (KRT19), and for IL1 family members IL1R-like 1 (IL1RL1) or IL33R, IL18R1, their respective ligands (IL33 and IL18) and both IL1 isoforms A and B.

**Table 2:** Genes up-regulated after in vitro treatment of human endometrium stromal cells.

Gene	*p*-value hCG/IL1	FC hCG/IL1	*p*-value MM/IL1	FC MM/IL1	*p*-value hCG/MM	FC hCG/MM	IPA Pf.	IPA Im.	IPA Rm.	IPA CS.	IPA Ap.	IPA Ag.
**CXCL6**	0.00001	35.52	0.00002	21.51				X		X		
**IL6^á^**	0.00088	15.06	0.00147	12.16			X	X	X	X	X	X
**CXCL1**	0.00067	13.94	0.00090	12.35			X	X		X	X	
**TNFAIP3**	0.00005	13.72	0.00027	8.03						X	X	X
**IL1B^á^**	0.00076	13.13	0.00020	23.61			X	X	X	X	X	
**CYP7B1**	0.00000	12.96	0.00001	5.09	0.02288	1.60	X		X			
**TNFAIP6**	0.00013	11.79	0.00448	4.08						X		
**PTGS2^á^**	0.00203	10.48	0.00368	8.33			X	X	X	X	X	
**CCL5^áâ^**	0.01045	9.57	0.03962	5.30			X	X		X	X	
**IL8**	0.00318	9.27	0.00227	10.59			X	X		X	X	
**C3**	0.00337	8.42	0.01599	4.83			X	X		X	X	
**VCAM1^á^**	0.00099	8.08					X	X		X		
**ITGB8**	0.00002	8.07	0.00079	3.39			X			X		
**CXCL2**	0.00077	7.25	0.00343	4.69			X			X	X	
**TNFAIP2**	0.00004	7.10										
**CCL2^áâ^**	0.00249	6.74	0.01108	4.25			X	X		X	X	
**CTSS**	0.00014	6.68	0.00064	4.57			X	X			X	
**IRAK3**	0.00001	6.56	0.00023	3.58						X	X	X
**CFB**	0.00235	6.14					X			X		
**EPSTI1**	0.00639	5.55										
**IL24**	0.00217	5.26	0.00063	7.59			X	X		X	X	
**IL13RA2**	0.01786	5.17	0.03330	4.14			X					
**MMP12**	0.01798	4.49	0.01483	4.79								
**NFKBIZ**	0.00003	4.14	0.00005	3.84							X	
**CX3CL1**	0.02049	3.95						X	X	X	X	
**IL7R**	0.00167	3.92	0.00162	3.95			X	X		X	X	X
**FGF7**	0.00367	3.87			0.02245	2.57	X	X	X	X		
**GNA15**	0.00250	3.85								X		
**CXCL3**	0.00228	3.81					X	X		X		
**CSF1**	0.00613	3.60					X	X	X	X	X	
**CCL8^á^**	0.00031	3.47						X		X		
**ZC3H12A**	0.00003	3.23	0.00010	2.76								
**ICAM1**	0.00001	3.13	0.00002	2.94			X	X	X	X	X	
**IL32**	0.00011	3.07	0.00285	1.97			X	X		X	X	
**ANK2**	0.00002	3.05			0.00215	1.73						
**CXCL5**	0.01034	3.02	0.01015	3.04			X			X		
**IL15RA**	0.00093	2.94					X	X		X	X	
**IL1A^á^**	0.00012	2.88	0.00001	4.43			X	X	X	X	X	
**PTGES**	0.00004	2.84					X	X		X	X	
**ITGA8**	0.01123	2.76										
**IRAK2**	0.01232	2.67	0.00250	3.75						X		X
**NFKBIA**	0.00003	2.60	0.00007	2.37			X			X	X	X
**WNT2**	0.00299	2.54			0.00760	2.20				X	X	
**LIF**	0.00019	2.37	0.00078	2.01			X	X	X	X	X	
**CYP1B1**	0.01872	2.35					X			X	X	
**BAMBI**	0.04533	2.28								X		
**IL1RL1^á^**	0.01807	2.05					X	X		X	X	
**FGF2**	0.00006	1.93	0.00006	1.93			X			X	X	
**HLA-DOB**	0.00425	1.89										
**C1R**	0.00242	1.83										
**CCL7^á^**	0.03055	1.80						X		X		

Gene	*p*-value hCG/IL1	FC hCG/IL1	*p*-value MM/IL1	FC MM/IL1	*p*-value hCG/MM	FC hCG/MM	IPA Pf.	IPA Im.	IPA Rm.	IPA CS.	IPA Ap.	IPA Ag.	
**C1S**	0.00019	1.79			0.00136	1.54							
**HLA-F**	0.00278	1.71											
**IL20**	0.00004	1.69					X			X			
**FOXP1**	0.00162	1.66					X			X	X		
**IL18R1^á^**	0.00483	1.65						X		X			
**IFNGR1**	0.01156	1.65					X			X	X		
**C2**	0.02976	1.65											
**SPAG1**	0.01701	1.64											
**IL7**	0.00011	1.63					X	X	X	X	X		
**TNFRSF1B**	0.00134	1.62					X	X	X	X	X		
**IL17RB**	0.01191	1.62						X					
**HLA-E**	0.00189	1.61					X						
**DUSP1**	0.01584	1.57					X	X		X	X		
**NFKB1**	0.00005	1.56					X	X	X	X	X		
**PDGFC**	0.00670	1.52					X			X			
**EGFR**	0.00112	1.51					X			X	X		
**CLDN12**	0.00231	1.50								?			
**INHBA**			0.03972	2.82			X	X		X	X		
**CLDN1**			0.00226	2.42						X			
**SERPINB3**			0.01389	2.09						X	X		X
**CYP19A1**			0.04113	2.05							X		
**VEGFC^áâ^**			0.03365	2.05			X			X	X		
**MMP9^áâ^**			0.00034	1.93			X		X	X	X		
**MMP3**			0.02941	1.81			X				X		
**IL4R**			0.00190	1.77			X	X		X	X		X
**SAT1**			0.01189	1.68			X				X		
**EPHA6**			0.01086	1.67									
**TRAF3IP2**			0.00848	1.63						X	X		
**NFKB2**			0.00022	1.62			X	X		X	X		
**MMP13**			0.01643	1.62					X	X			
**ADAM12**			0.02572	1.60						X	X		
**CASP3**			0.00748	1.59			X	X		X	X		
**ALDH1A1**					0.00743	4.10				X	X		
**CD34**					0.00212	2.14				X			
**FAT4**					0.00889	2.14							
**CXCL12**					0.04752	2.06	X	X	X	X	X		
**ANGPTL2**					0.01814	1.96							
**CLDN11**					0.01104	1.96	X			X			
**SEPP1**					0.02775	1.93				X			
**TMOD1**					0.00298	1.92				X			
**C1RL**					0.02441	1.67							
**CADM1**					0.01684	1.66	X			X	X		
**C4A**					0.04847	1.66		X		X			
**CRYAB**					0.04510	1.62	X				X		
**CNN1**					0.03209	1.60	X						
**SLC2A12**					0.00483	1.58							
**HSD17B6**					0.02452	1.55							
**SMAD9**					0.02996	1.54							
**ITPR1**					0.00157	1.53	X			X	X		X

Pf, proliferation; Im, immune functions; Rm, tissue remodeling; CS, cell signaling; Ap, apoptosis; Ag, angiogenesis

á/â Real-time PCR/ELISA validation performed

Genes up-regulated after in vitro treatment of human endometrium stromal cells

**Table 3:** Name of up-regulated genes.

RefSeq	Gene symbol	Genes name
NM_002993	CXCL6	chemokine (C-X-C motif) ligand 6
NM_000600	IL6	interleukin 6 (interferon, beta 2)
NM_001511	CXCL1	chemokine (C-X-C motif) ligand 1
NM_006290	TNFAIP3	tumor necrosis factor, alpha-induced protein 3
NM_000576	IL1B	interleukin 1, beta
NM_004820	CYP7B1	cytochrome P450, family 7, subfamily B, polypeptide 1
NM_007115	TNFAIP6	tumor necrosis factor, alpha-induced protein 6
NM_000963	PTGS2	prostaglandin-endoperoxide synthase 2 (prostaglandin G/H synthase and cyclooxygenase)
NM_002985	CCL5	chemokine (C-C motif) ligand 5
NM_000584	IL8	interleukin 8
NM_000064	C3	complement component 3
NM_001078	VCAM1	vascular cell adhesion molecule 1
NM_002214	ITGB8	integrin, beta 8
NM_002089	CXCL2	chemokine (C-X-C motif) ligand 2
NM_006291	TNFAIP2	tumor necrosis factor, alpha-induced protein 2
NM_002982	CCL2	chemokine (C-C motif) ligand 2
NM_004079	CTSS	cathepsin S
NM_007199	IRAK3	interleukin-1 receptor-associated kinase 3
NM_001710	CFB	complement factor B
NM_001002264	EPSTI1	epithelial stromal interaction 1 (breast)
NM_006850	IL24	interleukin 24
NM_000640	IL13RA2	interleukin 13 receptor, alpha 2
NM_002426	MMP12	matrix metallopeptidase 12 (macrophage elastase)
NM_031419	NFKBIZ	nuclear factor of kappa light polypeptide gene enhancer in B-cells inhibitor, zeta
NM_002996	CX3CL1	chemokine (C-X3-C motif) ligand 1
NM_002185	IL7R	interleukin 7 receptor
NM_002009	FGF7	fibroblast growth factor 7
NM_002068	GNA15	guanine nucleotide binding protein (G protein), alpha 15
NM_002090	CXCL3	chemokine (C-X-C motif) ligand 3
NM_000757	CSF1	colony stimulating factor 1 (macrophage)
NM_005623	CCL8	chemokine (C-C motif) ligand 8
NM_025079	ZC3H12A	zinc finger CCCH-type containing 12A
NM_000201	ICAM1	intercellular adhesion molecule 1
NM_001012631	IL32	interleukin 32
NM_001148	ANK2	ankyrin 2, neuronal
NM_002994	CXCL5	chemokine (C-X-C motif) ligand 5
NM_002189	IL15RA	interleukin 15 receptor, alpha
NM_000575	IL1A	interleukin 1, alpha
NM_004878	PTGES	prostaglandin E synthase
NM_003638	ITGA8	integrin, alpha 8
NM_001570	IRAK2	interleukin-1 receptor-associated kinase 2
NM_020529	NFKBIA	nuclear factor of kappa light polypeptide gene enhancer in B-cells inhibitor, alpha
NM_003391	WNT2	wingless-type MMTV integration site family member 2
NM_002309	LIF	leukemia inhibitory factor
NM_000104	CYP1B1	cytochrome P450, family 1, subfamily B, polypeptide 1
NM_012342	BAMBI	BMP and activin membrane-bound inhibitor homolog
NM_016232	IL1RL1	interleukin 1 receptor-like 1
NM_002006	FGF2	fibroblast growth factor 2 (basic)
NM_002120	HLA-DOB	major histocompatibility complex, class II, DO beta
NM_001733	C1R	complement component 1, r subcomponent
NM_006273	CCL7	chemokine (C-C motif) ligand 7
NM_201442	C1S	complement component 1, s subcomponent
NM_001098479	HLA-F	major histocompatibility complex, class I, F
NM_018724	IL20	interleukin 20
NM_032682	FOXP1	forkhead box P1
NM_003855	IL18R1	interleukin 18 receptor 1
NM_000416	IFNGR1	interferon gamma receptor 1
NM_000063	C2	complement component 2
NM_003114	SPAG1	sperm associated antigen 1
NM_000880	IL7	interleukin 7
NM_001066	TNFRSF1B	tumor necrosis factor receptor superfamily, member 1B
NM_018725	IL17RB	interleukin 17 receptor B

RefSeq		Gene symbol		Genes name
NM_005516	HLA-E		major histocompatibility complex, class I, E	
NM_004417	DUSP1		dual specificity phosphatase 1	
NM_003998	NFKB1		nuclear factor of kappa light polypeptide gene enhancer in B-cells 1	
NM_016205	PDGFC		platelet derived growth factor C	
NM_005228	EGFR		epidermal growth factor receptor	
NM_001185072	CLDN12		claudin 12	
NM_002192		INHBA		inhibin, beta A
NM_021101		CLDN1		claudin 1
NM_006919		SERPINB3		serpin peptidase inhibitor, clade B (ovalbumin), member 3
NM_031226		CYP19A1		cytochrome P450, family 19, subfamily A, polypeptide 1
NM_005429		VEGFC		vascular endothelial growth factor C
NM_004994		MMP9		matrix metallopeptidase 9
NM_002422		MMP3		matrix metallopeptidase 3 (stromelysin 1, progelatinase)
NM_000418		IL4R		interleukin 4 receptor
NR_027783		SAT1		spermidine/spermine N1-acetyltransferase 1
NM_001080448		EPHA6		EPH receptor A6
NR_028338		TRAF3IP2		TRAF3 interacting protein 2
NM_002502		NFKB2		nuclear factor of kappa light polypeptide gene enhancer in B-cells 2
NM_002427		MMP13		matrix metallopeptidase 13 (collagenase 3)
NM_003474		ADAM12		ADAM metallopeptidase domain 12
NM_004346		CASP3		caspase 3, apoptosis-related cysteine peptidase
NM_000689		ALDH1A1		aldehyde dehydrogenase 1 family, member A1
NM_001773		CD34		CD34 molecule
NM_024582		FAT4		FAT tumor suppressor homolog 4 (Drosophila)
NM_000609		CXCL12		chemokine (C-X-C motif) ligand 12
NM_012098		ANGPTL2		angiopoietin-like 2
NM_005602		CLDN11		claudin 11
NM_005410		SEPP1		selenoprotein P, plasma, 1
NM_003275		TMOD1		tropomodulin 1
NM_016546		C1RL		complement component 1, r subcomponent-like
NM_014333		CADM1		cell adhesion molecule 1
NM_007293		C4A		complement component 4A (Rodgers blood group)
NM_001885		CRYAB		crystallin, alpha B
NM_001299		CNN1		calponin 1, basic, smooth muscle
NM_145176		SLC2A12		solute carrier family 2 (facilitated glucose transporter)
NM_003725		HSD17B6		hydroxysteroid (17-beta) dehydrogenase 6 homolog (mouse)
NM_001127217		SMAD9		SMAD family member 9
NM_001168272		ITPR1		inositol 1,4,5-triphosphate receptor, type 1

Name of up-regulated genes.

**Table 4:** Genes down-regulated after in vitro treatment of human endometrium stromal cells.

Gene	*p*-value hCG/IL1	FC hCG/IL1	*p*-value MM/IL1	FC MM/IL1	*p*-value hCG/MM	FC hCG/MM	IPA Pf.	IPA Im.	IPA Rm.	IPA CS.	IPA Ap.	IPA Ag.
**ETV1**	0.02017	−5.26										
**HSD17B2**	0.03976	−5.06										
**LOXL4**	0.00528	−4.74	0.00435	−5.01								
**CDK1**	0.03192	−4.25					X				X	
**ITGA6**	0.00651	−3.82	0.01105	−3.35			X			X	X	
**DUSP6**	0.00051	−3.56			0.01223	−2.08				X	X	
**SLC20A1**	0.00306	−3.08								X		
**POLE2**	0.00645	−2.86			0.01939	−2.31						
**CCNA2**	0.02166	−2.66					X			X	X	
**MCM6**	0.00275	−2.54										
**KRT19^á^**	0.00000	−2.50	0.00001	−2.26								
**RFC3**	0.00785	−2.49										
**KRT34**	0.00247	−2.45										
**E2F7**	0.04905	−2.38					X					
**BRCA1**	0.01466	−2.25			0.02641	−2.04	X				X	
**PAK1**	0.00399	−2.24	0.02263	−1.77			X		X	X	X	
**HELLS**	0.04234	−2.22									X	
**ANGPTL4**	0.01955	−2.09					X				X	
**ITGA4**	0.00435	−2.03	0.00371	−2.08					X	X	X	X
**ACTA2**	0.01071	−1.91	0.02904	−1.68						X		
**CCND1**	0.00041	−1.90					X			X	X	
**HOXA11**	0.00279	−1.88	0.00047	−2.31								
**ITGA3**	0.00146	−1.69					X	X		X		
**FZD1**	0.00697	−1.58	0.00048	−2.06						X		
**POLA2**	0.02091	−1.51			0.00795	−1.66						
**ITGB5**			0.03518	−1.53			X			X	X	X
**ANXA4**			0.02022	−1.53							X	
**TIMP3^áâ^**			0.01839	−1.57			X			X	X	X
**BCL2L10**			0.00814	−1.58							X	
**SMAD9**			0.01991	−1.61								
**IGF2BP3**			0.02094	−1.62			X					
**C5**			0.01257	−1.68			X	X	X	X	X	
**TGFBR3**			0.04598	−1.82			X	X		X	X	
**ANGPTL2**			0.02817	−1.84								
**FAS**			0.03925	−1.85			X	X		X	X	X
**LAMB1**			0.03736	−1.91			X			X		
**TNFRSF19**			0.02307	−2.26							X	
**PDGFD**			0.01484	−3.19			X			X		
**MMP10^á^**					0.01030	−3.91					X	
**IL13RA2**					0.04783	−3.64	X					
**CCNE2**					0.01264	−2.78	X			X		
**PLAU**					0.00713	−2.42	X			X	X	X
**MELK**					0.01473	−2.41						
**ITGA2**					0.01329	−2.37	X			X	X	X
**CDC45**					0.01108	−2.30	X				X	
**SPAG5**					0.03517	−2.16	?					
**BRCA2**					0.03864	−1.96	X			X	X	
**E2F8**					0.02061	−1.85	X					
**MMP3**					0.02542	−1.84	X				X	X
**MCM5**					0.01884	−1.79						
**MCM3**					0.02412	−1.62						
**ORC1**					0.00293	−1.60						
**PCNA**					0.02189	−1.59	X				X	
**E2F1**					0.00984	−1.55	X			X	X	X
**IL1A^á^**					0.02429	−1.52	X	X	X	X	X	X
**FOSL1**					0.01092	−1.50	X			X	X	

á/â Real-time PCR/ ELISA validation performed

Pf, proliferation; Im, immune functions; Rm, tissue remodeling; CS, cell signaling; Ap, apoptosis; Ag, angiogenesis

**Table 5:** Name of down-regulated genes.

RefSeq	Gene symbol	Genes name
NM_004956	ETV1	ets variant 1
NM_002153	HSD17B2	hydroxysteroid (17-beta) dehydrogenase 2
NM_032211	LOXL4	lysyl oxidase-like 4
NM_001786	CDK1	cyclin-dependent kinase 1
NM_000210	ITGA6	integrin, alpha 6
NM_001946	DUSP6	dual specificity phosphatase 6
NM_005415	SLC20A1	solute carrier family 20 (phosphate transporter), member 20
NM_002692	POLE2	polymerase (DNA directed), epsilon 2 (p59 subunit)
NM_001237	CCNA2	cyclin A2
NM_005915	MCM6	minichromosome maintenance complex component 6
NM_002276	KRT19	keratin 19
NM_002915	RFC3	replication factor C (activator 1) 3, 38kDa
NM_021013	KRT34	keratin 34
NM_203394	E2F7	E2F transcription factor 7
NR_027676	BRCA1	breast cancer 1, early onset
NM_001128620	PAK1	p21 protein (Cdc42/Rac)-activated kinase 1
NM_018063	HELLS	helicase, lymphoid-specific
NM_139314	ANGPTL4	angiopoietin-like 4
NM_000885	ITGA4	integrin, alpha 4 (antigen CD49D, alpha 4 subunit of VLA-4 receptor)
NM_001141945	ACTA2	actin, alpha 2, smooth muscle, aorta
NM_053056	CCND1	cyclin D1
NM_005523	HOXA11	homeobox A11
NM_002204	ITGA3	integrin, alpha 3 (antigen CD49C, alpha 3 subunit of VLA-3 receptor)
NM_003505	FZD1	frizzled homolog 1 (Drosophila)
NM_002689	POLA2	polymerase (DNA directed), alpha 2 (70kD subunit)
NM_002213	ITGB5	integrin, beta 5
NM_001153	ANXA4	annexin A4
NM_000362	TIMP3	TIMP metallopeptidase inhibitor 3
NM_020396	BCL2L10	BCL2-like 10 (apoptosis facilitator)
NM_001127217	SMAD9	SMAD family member 9
NM_006547	IGF2BP3	insulin-like growth factor 2 mRNA binding protein 3
NM_001735	C5	complement component 5
NM_003243	TGFBR3	transforming growth factor, beta receptor III
NM_012098	ANGPTL2	angiopoietin-like 2
NM_000043	FAS	Fas (TNF receptor superfamily, member 6)
NM_002291	LAMB1	laminin, beta 1
NM_148957	TNFRSF19	tumor necrosis factor receptor superfamily, member 19
NM_025208	PDGFD	platelet derived growth factor D
NM_002425	MMP10	matrix metallopeptidase 10 (stromelysin 2)
NM_000640	IL13RA2	interleukin 13 receptor, alpha 2
NM_057749	CCNE2	cyclin E2
NM_002658	PLAU	plasminogen activator, urokinase
NM_014791	MELK	maternal embryonic leucine zipper kinase
NM_002203	ITGA2	integrin, alpha 2 (CD49B, alpha 2 subunit of VLA-2 receptor)
NM_001178010	CDC45	cell division cycle 45 homolog (S. cerevisiae)
NM_006461	SPAG5	sperm associated antigen 5
NM_000059	BRCA2	breast cancer 2, early onset
NM_024680	E2F8	E2F transcription factor 8
NM_002422	MMP3	matrix metallopeptidase 3 (stromelysin 1, progelatinase)
NM_006739	MCM5	minichromosome maintenance complex component 5
NM_002388	MCM3	minichromosome maintenance complex component 3
NM_004153	ORC1	origin recognition complex, subunit 1
NM_002592	PCNA	proliferating cell nuclear antigen
NM_005225	E2F1	E2F transcription factor 1
NM_000575	IL1A	interleukin 1, alpha
NM_005438	FOSL1	FOS-like antigen 1

**Figure 3:**
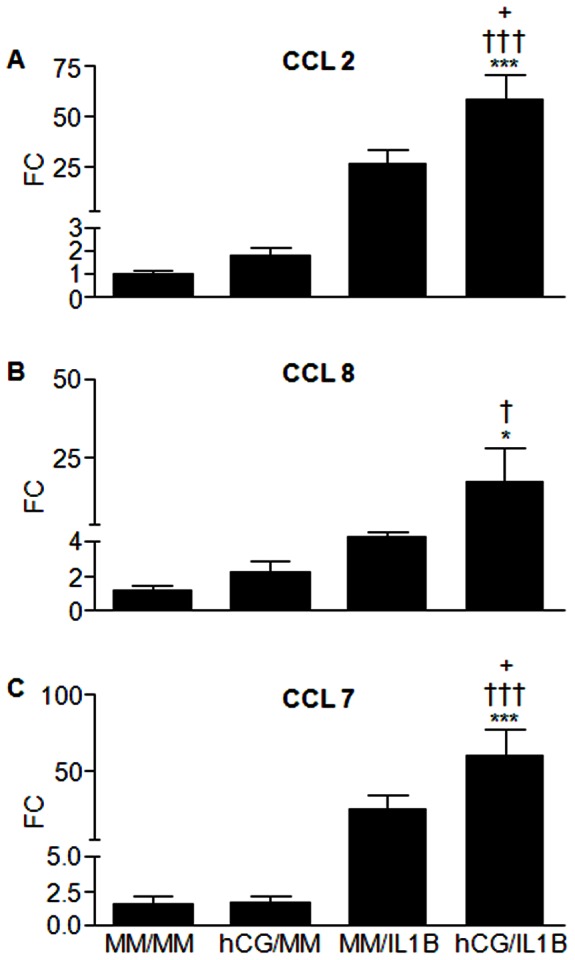
hCG modulates MCPs' mRNA expression in ESCs. Confluent ESC cultures were incubated with minimal medium (MM) (control) or hCG (100 ng/mL) for 24 h before being exposed or not to IL1B (0.1 ng/mL) for additional 24 h. Total RNA was extracted and reverse transcribed. CCL2, CCL8, CCL7 and GAPDH (internal control) mRNA levels were quantified by real-time PCR. CCL2 (A), CCL8 (B) and CCL7 (C) mRNA ratio was then determined following normalization to GAPDH mRNA. Data were from ESC cultures issued from 7 different subjects and expressed as fold change (FC) over control (ratio of CCL2, CCL8 or CCL7 mRNA levels found in cells incubated with IL1B, hCG or hCG/ILB to those found in cells incubated with MM for an equivalent period of time). *P<0.05, *** P<0.001 relative to MM; †P<0.05, †††P<0.001 relative to cells stimulated with an equivalent concentration of hCG; +P<0.05 relative to cells stimulated with an equivalent concentration of IL1B. Data were obtained with ESC cultures issued from 7 different subjects (the 3 cultures used for microarray analysis and 4 additional cultures).

Our previous studies showed that hCG acts on ESCs, in synergy with IL1B, to stimulate the expression of CCL2 (MCP1), a monocyte/macrophage chemotactic factor with potent angiogenic properties, *via* the creation of an imbalance between IL1R1 and IL1R2 expression [23]. Micro-array analysis and qRT-PCR validation indicated that not only CCL2, but CCL8 (MCP2) and CCL7 (MCP3) were significantly upregulated by hCG/IL1 as well (P<0.001, P<0.05 and P<0.001, respectively). Furthermore, a significant increase of CCL2, CCL8 and CCL7 mRNA transcripts in cells exposed to hCG/IL1B compared to hCG was noted (P<0.001, P<0.05 and P<0.001, respectively), whereas only CCL2 and CCL7 mRNA transcripts were significantly increased in cells treated with hCG/IL1B compared to IL1B (P<0.05) (Figure 3 A, B and C).

**Figure 4:**
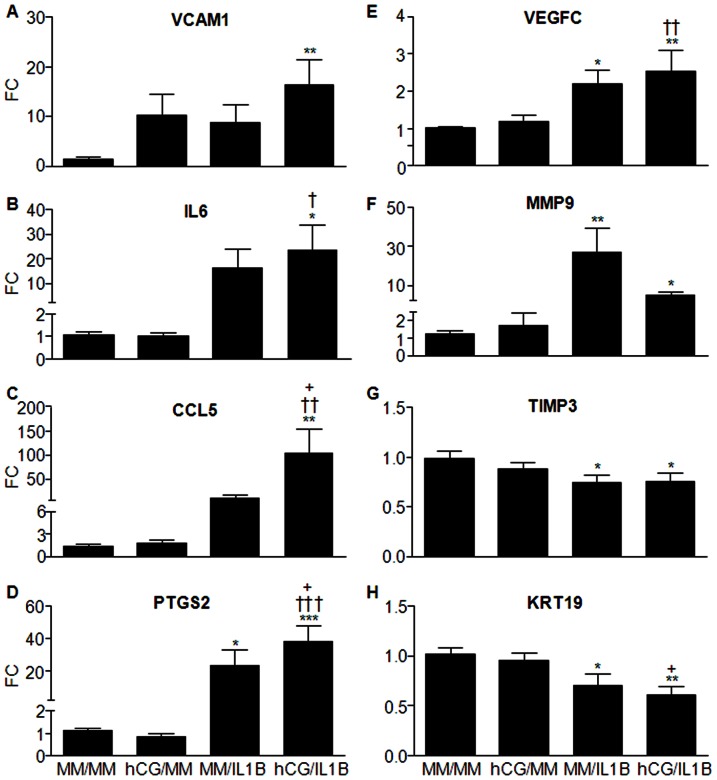
hCG modulates the IL1B-mediated mRNA expression of immune modulating, adhesion, growth, angiogenic and tissue remodeling factors in ESCs. Confluent ESC cultures were incubated with minimal medium (MM) (control) or hCG (100 ng/mL) for 24 h before being exposed or not to IL1B (0.1 ng/mL) for additional 24 h. Total RNA was extracted and reverse transcribed, and mRNA levels were then quantified by qRT-PCR. VCAM1 (A), IL6 (B), CCL5 (C), PTGS2 (D), VEGFC (E), MMP9 (F), TIMP3 (G) and KRT19 (H) mRNA ratio was then determined following normalization to GAPDH mRNA (internal control). Data were from ESC cultures issued from 7 different subjects and expressed as fold change (FC) over control (ratio of VCAM1, IL6, CCL5, PTGS2, VEGFC, MMP9, TIMP3 or KRT19 mRNA levels found in cells incubated with IL1B, hCG or hCG/ILB to those found in cells incubated with MM for an equivalent period of time). *P<0.05, **P<0.01, *** P<0.001 relative to MM; †P<0.05, ††P<0.01, †††P<0.001 relative to cells stimulated with an equivalent concentration of hCG; +P<0.05 relative to cells stimulated with an equivalent concentration of IL1B. Data were obtained with ESC cultures issued from 7 different subjects (the 3 cultures used for microarray analysis and 4 additional cultures).

Many other cytokines and growth factors known for being involved in the regulation of immune responses, adhesion, cell proliferation and angiogenesis were also found to be targeted by hCG and IL1B synergistic action. Vascular cell adhesion protein 1, which mediates leukocyte adhesion to vascular endothelium, was significantly up-regulated by hCG/IL1B compared to the control minimal medium (MM) (P<0.01), but neither hCG nor IL1B had a statistically significant stimulatory effect (Figure 4A). IL6 and CCL5 mRNA levels were significantly increased by hCG/IL1B compared to MM (P<0.05 and P<0.01, respectively) or to hCG (P<0.05 and P<0.01, respectively). CCL5 levels were also significantly increased in cells treated with hCG/IL1B compared to IL1B (P<0.05) (Figure 4B, C).

PTGS2, a major rate-limiting enzyme involved in PG synthesis, and VEGFC, an isoform of a potent angiogenic factor, also showed an increased mRNA expression in cells treated with hCG/IL1B compared to cells incubated with MM (P<0.001 and P<0.01, respectively) or with hCG alone (P<0.001 and P<0.01, respectively). IL1B stimulated PTGS2 and VEGFC mRNA synthesis as well (P<0.05), but cell exposure to hCG significantly stimulated in the IL1B-induced PTGS2 expression (P<0.05) (Figure 4D, E).

Also found to be regulated were some molecules shown to participate in endometrial tissue remodeling such as MMP9, TIMP3 and KRT19 (Figure4F, G, H). MMP9 was significantly up-regulated by IL1B and hCG/IL1B (P<0.01 and P<0.05, respectively). By itself, hCG had no statistically significant effect on MMP9 expression, but it moderated the IL1B-induced effect. TIMP3, a natural tissue inhibitor of MMPs, was down-regulated by IL1B either in the presence or the absence of hCG (P<0.05). Cytokeratin 19 (KRT19), an intermediate filament protein associated with embryonic placenta development [54], was found to be significantly inhibited by IL1B (P<0.05) and more by IL1B combined with hCG (P<0.01).

### hCG modulates IL1B effects on the expression of IL1 family members in ESCs

**Figure 5:**
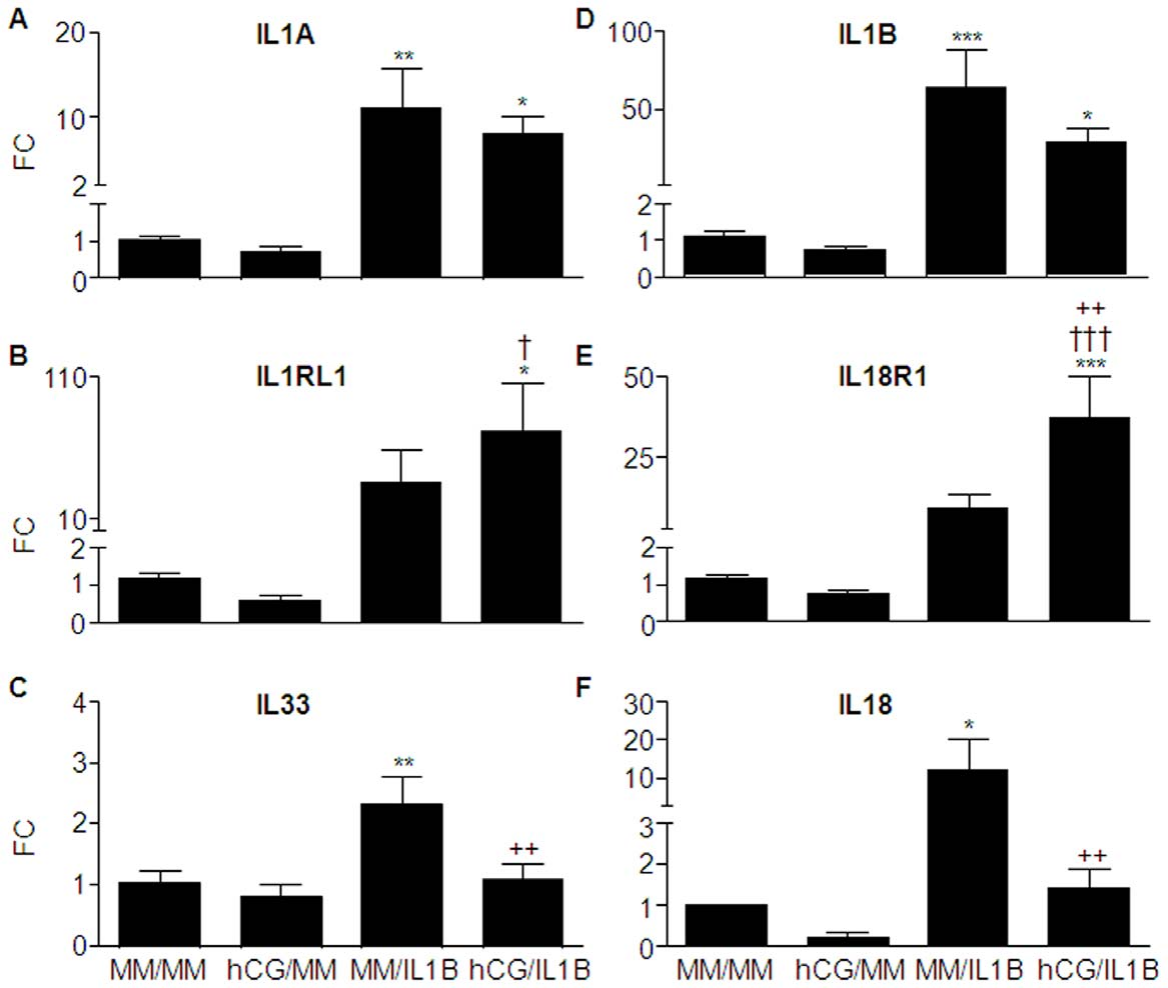
hCG modulates IL1B effects on the expression of IL1 family members in ESCs. Confluent ESC cultures were incubated with minimal medium (MM) or hCG (100 ng/mL) for 24 h before being exposed or not to IL1B (0.1 ng/mL) for additional 24 h. Total RNA was extracted and reverse transcribed, and mRNA levels were then quantified by qRT-PCR. IL1A (A), IL1B (D), IL1RL1 (B), IL18R1 (E), IL33 (C) and IL18 (F) mRNA ratio was then determined following normalization to GAPDH mRNA (internal control). Data were from ESC cultures issued from 7 different subjects and expressed as fold change (FC) over control (ratio of IL1A, IL1B, IL1RL1, IL18R1, IL33 or IL18 mRNA levels found in cells incubated with IL1B, hCG or hCG/ILB to those found in cells incubated with MM for an equivalent period of time). *P<0.05, **P<0.01, *** P<0.001 relative to MM; †P<0.05, †††P<0.001 relative to cells stimulated with an equivalent concentration of hCG; ++P<0.01 relative to cells stimulated with an equivalent concentration of IL1B. Data were obtained with ESC cultures issued from 7 different subjects (the 3 cultures used for microarray analysis and 4 additional cultures).

Our previous studies showed that hCG down-regulates the IL1B-induced increase of IL1R2 and IL1RN in ESCs and amplifies the IL1B-induced increase of IL1R1 [23]. Our current micro-array analysis further revealed that hCG/IL1B interaction may affect ESC responsiveness to other components of IL1 family including IL1 isoforms A and B as well as IL1RL1 and IL18R1 (Tables 2 and 4). To validate these findings, qRT-PCR analysis was performed. Our results showed that IL1B induced both ILA and IL1B in ESCs (P<0.01 and P<0.001, respectively), but hCG moderated that endogenous IL1B-mediated expression (Figure 5 A, D). However, both IL1 isoforms remained significantly up-regulated compared to the control medium (P<0.05) despite the down-regulatory effect of hCG. Furthermore, the micro-array data predicted a synergistic interaction between hCG and IL1B, resulting in the induction of IL18R1 and IL1R1L expression (Figure 5 B, E). This was confirmed by qRT-PCR analysis, which showed a significant up-regulation of these two receptors as compared to control (P<0.05 and P<0.001, respectively) and to hCG (P<0.05 and P<0,001, respectively). However, despite a noticeable increase of IL1RL1 and IL1R18 in hCG/IL1B-treated compared to IL1B-treated cells, this increase was statistically significant only for IL18R1 (P<0.01), but did not reach statistical significance for IL1RL1 with these small groups.

Because IL18R1 and IL1RL1 were targeted by hCG/IL1B, we have then investigated their ligands (IL18, IL33) by qRT-PCR. Our data showed that like IL1A and IL1B, both IL33 and IL18 were up-regulated in cells treated with IL1B (P<0.01 and P<0.05, respectively), but they were down-regulated in cells treated with hCG and IL1B compared to IL1B alone (P<0.01) (Figure 5 C, F).

### Validation of selected soluble proteins

**Figure 6:**
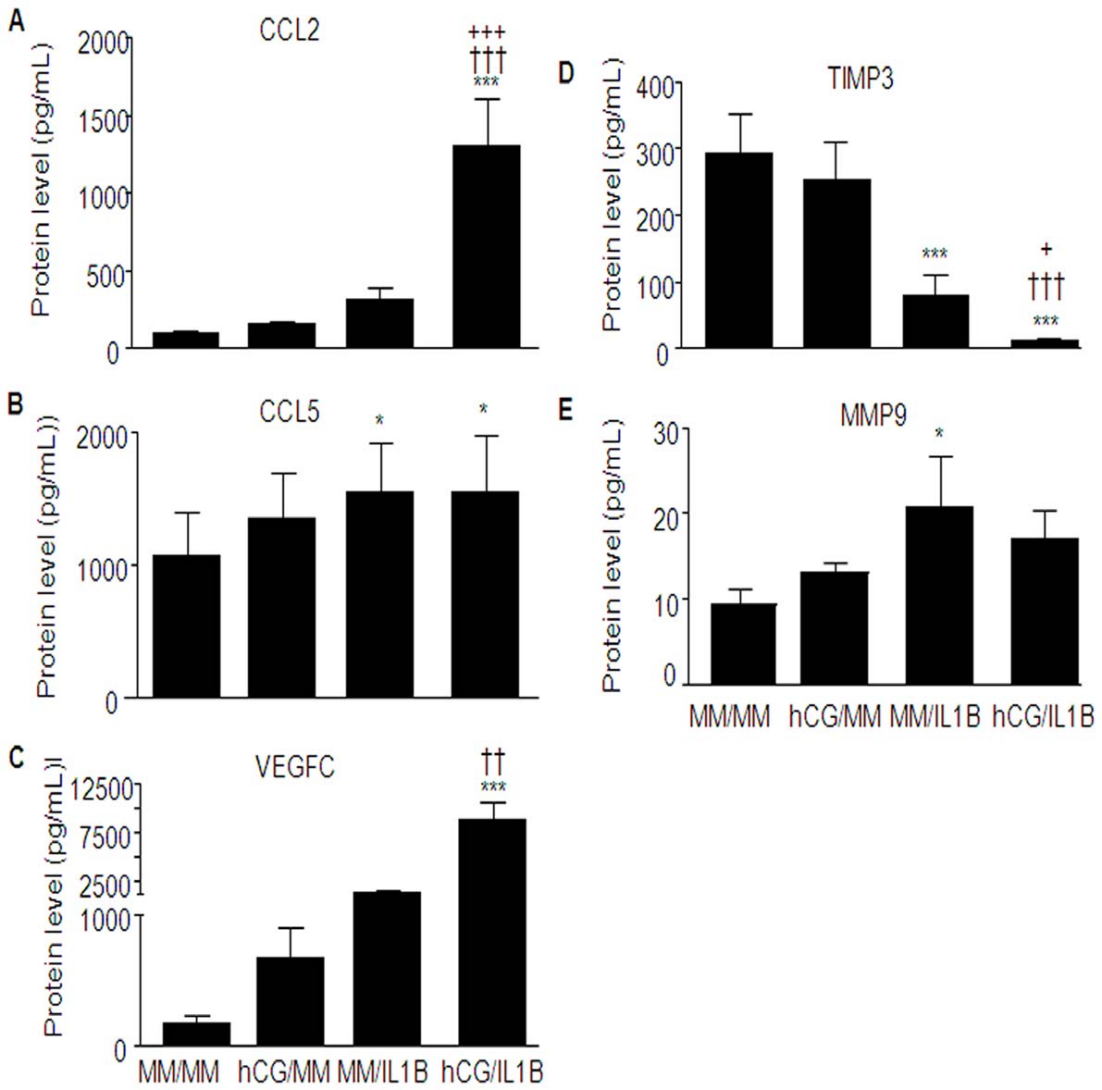
hCG modulates the expression of immune, angiogenic and tissue remodeling factors in ESCs at the protein level. Confluent ESC cultures were incubated with minimal medium (MM) or hCG (100 ng/mL) for 24 h before being exposed or not to IL1B (0.1 ng/mL) for additional 24 h. Supernatant was collected and soluble proteins CCL2 (A) CCL5 (B) VEGFC (C) TIMP3 (D) MMP9 (E) were then quantified by ELISA. Data were from ESC cultures issued from 4 different subjects and expressed in pg/mL. *P<0.05, *** P<0.001 relative to MM; ††P<0.01, †††P<0.001 relative to cells stimulated with an equivalent concentration of hCG; +P<0.05, +++P<0.001 relative to cells stimulated with an equivalent concentration of IL1B.

To confirm the gene expression changes at the protein level, we have selected some genes found to be significantly regulated upon hCG, IL1B or hCG/IL1B treatment and known for being involved in immune modulation, tissue remodeling and angiogenesis. Results indicated that CCL2 was significantly upregulated by hCG/IL1B (P<0.001), which corroborates the microarray data (Figure 6A). CCL5 secretion was upregulated either by IL1B or hCG/IL1B (P<0.05), but hCG/IL1B synergism was not perceptible at the protein level (Figure 6 B). hCG/IL1B treatment further appeared to upregulate VEGFC (P<0.001) and downregulate TIMP3 secretion (P<0.001) (Figure 6 C, D). MMP9 was significantly up-regulated by IL1B (P<0.05), but hCG/IL1 did not show a statistically significant stimulatory effect (Figure 6E).

## Discussion

Cumulating evidences point to an important role for embryo-endometrial dialogue in mediating the adhesion, invasion and growth of the embryo during implantation and early development. Well-known for rescuing the corpus luteum and maintaining the production of progesterone, embryo-derived signals such as hCG seem to orchestrate endometrial adaptation to the implantation of the newly formed embryo as well [22], but little is known about the involved pathways and the underlying mechanisms. Human CG is quite known, for instance, for stimulating cytokine/chemokine production by endometrial epithelial and stromal cells and playing direct and indirect roles in human ESC decidualization [17] and angiogenesis in human endothelial cells [19], [23], [55]. It is unclear if hCG is involved in blastocyst attachment in humans. However, it may have an indirect role, as suggested by our and other studies, and act via the modulation of human epithelial cell receptivity/responsiveness to other major embryonic signals such as IL1 [56]. A recent study demonstrated that the expression of β3-integrin-subunit, a cell adhesion mediator and marker of uterine receptivity [57], on the surface of human endometrial epithelial cells could be up-regulated by coculture with a human preimplantation embryo and blocked by anti-IL1 antibody [35]. Another study showed that the expression of trophinin, which mediates cell adhesion by homophilic binding, and the ability for apical cell adhesion with trophinin-expressing human trophoblastic cells are increased in presence of hCG associated with IL1β [58].

The presence of LHCG receptor in various endometrial cell types [15], [16] makes plausible that hCG has a broad spectrum of endometrial cell targets. After the intrusion of the embryo through the luminal endometrial epithelium, trophoblastic cells are in close contact with different maternal stromal cell types [59]. To achieve a successful pregnancy, an appropriate cross-talk between embryonic and maternal cells must therefore take place, where numerous embryo- as well as maternal-derived factors including steroid hormones, matrix degrading enzymes, integrins, cytokines, chemokines and growth factors could be involved [60], [61].

Our previous studies revealed a new mechanism by which hCG can target different human endometrial cell types, including epithelial and stromal cells, to modulate their receptivity to IL1, an early potent embryonic signal, and amplify thereby the release of immune and angiogenic factors [23], [42]. In the present study, we further showed that hCG acts on ESCs, either alone or *via* the modulation of the IL1-mediated cell responsiveness, to regulate numerous relevant genes involved in cell signaling, proliferation, apoptosis, immune modulation, tissue remodeling and angiogenesis, which are highly relevant mechanisms underlying the implantation process and the modulation of the immune response around the implanting embryo. Some of the genes were known for playing important roles in the various embryo implantation stages, but many genes were not known for being possibly involved and regulated by hCG or hCG/IL1B synergism.

MCPs were among the most significantly induced immune factors in ESCs in response to hCG and IL1B. hCG amplified the IL1B-induced expression of MCP1, 2 and 3. These chemokines are involved in the recruitment of monocytes/macrophages, T cells and NK cells into inflammatory sites [62], [63]. Moreover, MCPs stimulate angiogenesis, either directly *via* MCP1-induced protein (MCPIP) or indirectly *via* their activation of immune cells such as NK cells and macrophages, which are known for releasing growth and angiogenic factors [64]. Interestingly, the current micro-array data are in keeping with our previous findings of an increased IL1B-mediated secretion of MCP1 in human ESCs from the implantation window following hCG treatment [23] and consistent with a possible role for MCPs in embryo implantation. Actually, macrophages contribute to decidualization and implantation and remain abundant at the implantation site throughout pregnancy [65], [66], and trophoblastic cells were shown to regulate human monocyte migration and differentiation [67]. However, uterine macrophages do not appear to impair the growth of the semi-allogeneic embryo. They rather seem to play a protective role against possible infections, maintain immune tolerance toward trophoblastic antigens, mediate trophoblast invasion and support embryonic growth [68]. This strengthens the relevance of our findings and broadens the spectrum of hCG's impact on early embryonic growth and development.

VCAM1, an adhesion molecule of endothelial cells playing an important role in immune cell trafficking [69], appeared to be up-regulated by hCG or IL1B, but significantly by hCG and IL1B in ESCs. During pregnancy, the few available reports suggest a possible role for VCAM1. The expression of this adhesion molecule is strongly induced in the endothelium of early pregnant sheep endometrium [70] and decreases in fetal membranes with advancing gestational age [71]. However, its role in human pregnancy and during embryo implantation remains to be elucidated.

Many other cytokines including IL6, CCL5 (RANTES) and VEGFC appeared to be targeted by hCG and IL1B synergistic action. These pluripotent factors are quite known for being involved in the regulation of immune response, cell proliferation, tissue remodeling and angiogenesis. First identified as a promoter of B-cell differentiation and antibody production, IL6 is nowadays known as a pleiotropic cytokine that regulates cell growth, angiogenesis, inflammation and hematopoiesis [72]. IL6 expression was described in human granulosa and theca cells, endometrium and pre-implantation embryo [73]. Also, habitual abortion in women is associated with a decrease in expression of IL1B and IL6 [74], suggesting a role for these cytokines in the maintenance of pregnancy. A growing body of evidence implicates CCL5 in the induction of tolerance at immune-privileged sites. This cytokine seems to suppress maternal allogeneic responses, which is necessary for successful implantation [75], [76], [77], [78]. VEGFC is primarily a potent angiogenic growth factor and may play an important role in embryonic cell growth. However, it was described recently as an immune modulator that induces immune tolerance in murine tumor cells [79]. Therefore, these hCG/IL1B-induced biological properties in endometrial cells may represent a relevant mechanism involved in the immune tolerance of the implanting embryo within the uterine maternal host. This is in keeping with a previous study reporting that *in vivo* infusion of IL1B and hCG induces endometrial changes that mimic early pregnancy events in the baboon and lead to the development of an immunotolerant environment [80].

Invasion of the trophoblast into the endometrium requires a delicate balance between tissue degradation and maintenance. Up-regulation of MMP9 expression in endometrial cells by IL1B in human endometrial cells [81] and secretion by cultured first trimester human trophoblastic cells and fibroblasts has been demonstrated [82]. In addition, the expression of MMPs correlated with the invasive potential of human trophoblast cells [82]. Our micro-array data and qRT-PCR validation revealed the regulation of several tissue remodeling mediators such as MMP9, TIMP3 and KRT19. MMP9 was significantly up-regulated by IL1B in ESCs and hCG seemed to moderate this action, which, however, remained significant compared to non-stimulated cells. TIMP3, a natural tissue inhibitor of MMPs [83] was down-regulated by IL1B either in the presence or the absence of hCG. The reduction of TIMP3 expression levels, combined with the increased expression of MMP9, may create an imbalance that favors tissue matrix proteolysis and embryo implantation. However, the recent literature reporting that TIMP3 induces apoptosis, inhibits angiogenesis and impedes cell migration [84] makes highly relevant our present findings, considering the crucial importance of embryonic cell survival, proliferation and migration for the establishment of early pregnancy. Interestingly, KRT19, a molecule associated with embryonic placenta development [54] was found to be significantly inhibited by IL1B and more by IL1B combined with hCG. KRT19 is an intermediate filament protein and an epigenetically regulated tumor suppressor gene down-regulated in several cancerous tumors [85]. Also, down-regulation of KRT19 in human oral squamous cell carcinoma lines increases the invasive potential [86], but no other previous studies showed any eventual relationship with the invasive capacity of embryonic cells.

Micro-array and qRT-PCR validation data further revealed a synergistic interaction between hCG and IL1B to induce PTGS2, which is a rate limiting enzyme for PG synthesis. This is quite relevant considering the well-documented role of PGs as key regulators of female reproductive tract functions, including ovulation, menstruation and myometrial contractility, and vascular permeability and angiogenesis at the implantation site [87], [88], [89].

Interestingly, validation of the expression of some major selected genes at the protein level corroborates the combined role of hCG and IL1B in the modulation of angiogenic, immune and tissue remodeling functions of ESCs. Actually, assessment of protein secretion showed that hCG and IL1B synergistically induced CCL2 and VEGFC, inhibited TIMP3 and moderate the IL1B-induced MMP9.

Our previous studies showed that hCG down-regulates the IL1B-induced increase in IL1R2 and IL1RN in human ESCs and further enhances the IL1B-induced increase in IL1R1, thereby amplifying *in vitro* the release of angiogenic activity [23]. Surprisingly, the results of the current micro-array analysis showed a broader spectrum of regulation encompassing other IL1 family members, and suggest a possible modulation of endometrial cell responsiveness to IL1 family. In fact, hCG appeared to potentiate the IL1B-induced expression of IL1R1L (IL33R) and IL18R and to moderate, on the other hand, the expression of endogenous IL33, IL18, IL1A and IL1B in ESCs. Nonetheless, the expression of IL1 isoforms was still up-regulated despite the down-regulatory effect of hCG. These results indicate a possible mechanism by which hCG may induce immunotropism and prevent undue local expression of proinflammatory cytokines, as excessive production levels may be associated with repeated miscarriage and fetal growth retardation [90].

It is noteworthy that according to the recent literature, hCG has been shown to be produced by human endometrial epithelial cells in the luteal phase [12]. Indeed, endogenous endometrial hCG may, though produced at low quantities, have a role in in embryo implantation and the hCG-mediated growth promoting effects, but this is still to be demonstrated.

In conclusion, our study showed that hCG induces major changes in human ESC phenotype and deeply modulates their responsiveness to a proinflammatory, but a growth mediator and a potent embryonic signal such as IL1B. Generally *via* synergistic stimulatory or inhibitory mechanisms, hCG induces significant alterations in the expression of genes known for being involved or having the potential to play an important role in embryonic implantation and growth and the modulation of the immune response around the implanting blastocyst. Furthermore, our study revealed that the modulation of endometrial cell receptivity *via* hCG is not limited to IL1 receptors' agonists and antagonists, but also extends to other IL1 family members, which share numerous growth-promoting, immune-modulating and signaling pathways. This, together with our previous data showing that hCG can similarly target different endometrial cell types, strengthens the relevance of such a modulatory mechanism for implantation and early embryonic growth within the host maternal endometrial tissue and further suggests that hCG plays an important role in the establishment of a receptive endometrial phenotype.
